# FOXO4-DRI regulates endothelial cell senescence via the P53 signaling pathway

**DOI:** 10.3389/fbioe.2025.1729166

**Published:** 2026-01-15

**Authors:** Zhicheng Hu, Fan Li, Chunyi Hu, Qiongdan Shan, Zhouhao Tang, Meifan Jiang, Xiaojing Yi, Xixi Chen, Litai Jin, Xu Wang, Yang Wang

**Affiliations:** 1 School of Pharmaceutical Science, Wenzhou Medical University, Wenzhou, China; 2 Oujiang Laboratory (Zhejiang Lab for Regenerative Medicine, Vision and Brain Health), School of Pharmaceutical Science, Wenzhou Medical University, Wenzhou, China; 3 Department of Histology and Embryology, School of Basic Medical Sciences, Wenzhou Medical University, Wenzhou, China; 4 Department of Pharmacy, Taizhou Central Hospital, Taizhou, Zhejiang, China; 5 Ningbo Key Laboratory of Skin Science, Ningbo College of Health Sciences, Ningbo, China

**Keywords:** D-Gal, endothelial cell senescence, FOXO4-DRI, P53, ROS

## Abstract

**Objectives:**

Endothelial cell dysfunction during aging is a key driver of vascular aging and related diseases; however, effective strategies to selectively eliminate senescent endothelial cells and restore vascular function remain lacking. FOXO4-DRI, a novel peptide-based intervention, specifically disrupts the interaction between FOXO4 and P53, thereby inducing apoptosis in senescent cells. This study innovatively focuses on the mechanism by which FOXO4-DRI induces apoptosis in senescent endothelial cells, demonstrating that it functions by activating the p53/BCL-2/Caspase-3 signaling pathway to promote selective apoptosis of these cells. FOXO4-DRI significantly improves vascular function and delays vascular aging. These findings not only enrich the molecular understanding of senescent cell clearance but also provide a novel strategy for precise targeting of endothelial cell senescence in therapeutic applications.

**Materials and methods:**

This study aims to analyze the vascular function and aging status of the aorta in naturally aged mice and progeroid model mice following FOXO4-DRI injection. Additionally, it investigates changes in endothelial cell function in senescent endothelial cells induced by oxygen-glucose deprivation (OGD), as well as the protein expression and interaction in the FOXO4-P53 signaling pathway. To assess the impact of FOXO4-DRI on endothelial cell senescence, the senescent endothelial cells were treated with FOXO4-DRI, followed by immunofluorescence and Western blotting experiments.

**Results:**

Injection of FOXO4-DRI in both naturally aged and induced aging mice effectively suppressed aortic aging and improved aortic function. Additionally, we found that FOXO4-DRI alleviates endothelial cell senescence induced by OGD, thereby enhancing endothelial cell function. Through co-immunoprecipitation (CO-IP) experiments, we discovered that FOXO4-DRI prevents the binding of FOXO4 to P53, facilitating the phosphorylated P53 nuclear exclusion, which subsequently trigger BAX and cleaved caspase-3, leading to the apoptosis of senescent cells. Ultimately, this mechanism achieves the goal of inhibiting vascular aging.

**Conclusion:**

FOXO4-DRI promotes the nuclear export of phosphorylated P53 by inhibiting the binding of FOXO4 to P53 in endothelial cells, thereby facilitating the apoptosis of senescent endothelial cells and alleviating aging.

## 1 Introduction

Aging has always been one of the significant concerns for humans. In recent years, with the advancement of technology, people have conducted more in-depth research into the causes of aging. Fundamentally, aging is driven by the cumulative effects of cellular senescence (Arianna et al., 2019). Research indicates that cellular senescence refers to the loss of cell division and normal functional capabilities, making it the basic unit of biological aging (Barnes et al., 2019). It is worth noting that cellular senescence also plays important beneficial roles in physiological processes such as tissue development, wound healing, and tumor suppression (Suter et al., 2025). The process of cellular senescence involves multiple factors, including DNA damage, telomere shortening, and epigenetic changes, which lead to cell cycle arrest and ultimately result in cellular senescence (Zhang et al., 2024). Additionally, under stress or disease conditions, such as hypoxia, cellular senescence can be induced. Particularly in vascular endothelial cells, hypoxia accelerates endothelial cell senescence, significantly affecting vascular dilation function (Gao et al., 2024; Avrutsky et al., 2023). This functional disorder not only disrupts the normal regulatory mechanisms of blood vessels but also leads to increased blood pressure, arterial stiffness, and remodeling of small arteries, ultimately resulting in multi-organ damage (Boutouyrie et al., 2021; Véronique et al., 2024). Furthermore, the impact of endothelial cell senescence is not limited to the vascular system. By inducing vascular dysfunction, inflammation, and oxidative stress, it becomes a key driver of aging in multiple organs such as the heart, brain, and kidneys. For example, the senescence of cardiac endothelial cells may exacerbate myocardial ischemia and heart failure. At the same time, endothelial cell senescence also affects the delivery of nutrients and oxygen, leading to metabolic abnormalities and further accelerating organismal aging (Boutouyrie et al., 2021; Véronique et al., 2024).

The strategies to address senescent cells and replenish exhausted stem cells during the aging process, as well as supplementing metabolic substances like NAD+, gene therapy, or epigenetic approaches to mitigate aging, are different because they aim to promote the repair of senescent cells rather than inducing their apoptosis (Covarrubias et al., 2020; Feinberg Andrew et al., 2023; Birch and Jesús, 2020). However, senescent cells cause more damage to the body than we initially anticipated. These cells secrete large amounts of pro-inflammatory factors, triggering a localized or systemic low-grade chronic inflammatory response. This inflammatory state not only damages surrounding healthy cells but can also lead to excessive activation of the immune system, further exacerbating tissue damage (Byrns China et al., 2024; Prata et al., 2018). Additionally, senescent cells produce matrix metalloproteinases (MMPs) and other proteolytic enzymes that disrupt the extracellular matrix, interfering with normal tissue structure and function. Over time, this accumulation can lead to organ dysfunction. Therefore, clearing senescent cells is crucial for delaying the aging process (Su et al., 2021).

The Forkhead box O (FOXO) proteins are an important subclass of the Forkhead family. In mammals, this subclass is comprised of four main members: FOXO1, FOXO3, FOXO4, and FOXO6 (Santos Bruno et al., 2023). The FOXO family is involved in regulating critical processes such as stress resistance, metabolism, cell cycle arrest, and apoptosis (Martins et al., 2016). Specifically, FOXO activity is essential for the execution of apoptosis under stress conditions (Tomohiro and Sakamoto, 2008). Moreover, studies have indicated that the interaction between FOXOs and P53 can modulate aging in yeast and invertebrates (Chen et al., 2022). Therefore, FOXOs play a crucial role in regulating cellular aging and apoptosis. However, literature on the regulation of vascular aging by FOXOs is relatively limited.

Senescent cells typically exhibit enhanced resistance to apoptosis (Clara et al., 2024). In previous studies, FOXO4 and p53 have been shown to interact, a process that inhibits the pro-apoptotic role of p53 by restraining its nuclear exclusion, thereby promoting cell survival. It has been demonstrated that FOXO4-DRI can directly and with high affinity binds to the transactivation domain 2 (TAD2) of p53, competitively disrupting the FOXO4-p53 interaction within the nucleus. This leads to the release and subsequent cytoplasmic translocation of p53, which in turn activates a transcription-independent pro-apoptotic pathway, facilitating the selective clearance of senescent cells (Kong et al., 2025; Bourgeois et al., 2025). However, the efficacy of this strategy in the context of vascular aging remains to be elucidated, prompting our further investigation in this area.

Accordingly, in this study, we investigated the therapeutic potential of FOXO4-DRI in vascular aging. We demonstrated that in senescent vascular endothelial cells, FOXO4-DRI effectively disrupts the FOXO4-p53 interaction. This disruption initiates a critical sequence of events: it promotes the phosphorylation of p53 and facilitates its translocation to the cytoplasm. The liberated p53 then transcription-independently activates the pro-apoptotic protein BAX, thereby triggering the execution phase of apoptosis. Ultimately, this FOXO4-DRI-mediated mechanism selectively eliminates senescent endothelial cells, which in turn attenuates vascular inflammation and improves endothelial-dependent vasodilation, conferring a significant protective effect on vascular function.

## 2 Methods

### 2.1 Animals and procedures

The animal experimental procedures were approved by the Animal Care and Use Committee of Oujiang Laboratory (SYXK2023-0047, OJLAB22121609), and were carried out in strict accordance with the National Institutes of Health Guidelines for the Use of Laboratory Animals. The experimental mice were housed under standard conditions with an environmental temperature of 23 °C ± 2 °C and humidity of 50% ± 5%. The mice were randomly divided into four groups (each containing at least five mice): a natural aging group, a natural aging + FOXO4-DRI group, a D-Gal group, and a D-Gal + FOXO4-DRI group. The natural aging model was established in randomly selected male and female mice. At 17 months of age, the natural aging group received intraperitoneal injections of PBS every 2 days for 1 month, after which tissues were collected. In the natural aging + FOXO4-DRI group, mice received intraperitoneal injections of FOXO4-DRI (5 mg/kg, MCE, HY-P4157) every 2 days during the same period, followed by tissue collection. We established a D-galactose-induced aging model in randomly selected male and female mice through intraperitoneal injections of D-galactose (200 mg/kg/day, MCE, HY-N0210), starting at 8 weeks of age for 8 weeks. After 4 weeks of D-galactose injections, the D-Gal group mice received intraperitoneal injections of PBS every 2 days for 4 weeks, followed by tissue collection. In the D-Gal + FOXO4-DRI group, mice also received D-galactose injections and, after 4 weeks, were given intraperitoneal injections of FOXO4-DRI (5 mg/kg, MCE, HY-P4157) every 2 days for 4 weeks, followed by tissue collection. All mice were euthanized by isoflurane overdose. The animals were placed in an induction chamber with an oxygen-enriched environment, with the initial induction concentration set at 4%–5% isoflurane, until deep anesthesia signs such as loss of the righting reflex appeared, after which maintenance was initiated. Subsequently, isoflurane was maintained at 5%–6% in the same exposure system to achieve overdose and terminate life promptly. Death typically occurred within 3–10 min after the start of exposure, with the exact duration influenced by factors such as body weight, age, room ventilation, and equipment. Death was determined based on standardized criteria including the absence of respiration, absence of heartbeat, loss of pupillary light reflex, and absence of reflexes, with scavenging and operator protection measures in place.

### 2.2 Culture and treatment of human umbilical vein endothelial cells (HUVECs)

HUVECs were purchased from Shanghai Zhong Qiao Xin Zhou Biotechnology Co., Ltd. (PCM-H-040, ZQXZbio, China). All cells were cultured in an endothelial cell-specific medium for human umbilical vein endothelial cells, which was supplemented with 10% fetal bovine serum and 1% penicillin/streptomycin (PCM-H-040, ZQXZbio, China). The cells were maintained in a humidified incubator at 37 °C and 5% CO_2_. For the FOXO4-DRI treatment group, 50 μM FOXO4-DRI (MCE, HY-P4157) was added to the glucose-free DMEM (Gibco, Catalog #11966-025) medium, followed by 3 h of oxygen-glucose deprivation (OGD) treatment. For the control group, after the same initial treatment, the FOXO4-DRI was replaced with an equal volume of PBS.

### 2.3 Western blot and antibodies

The equal amounts (30 μg) of protein lysed from aortic tissue and HUVECs, were separated by SDS-PAGE and then transferred onto polyvinylidene fluoride (PVDF) membranes (Merck Millipore, IPVH00010). Next, membranes were blocked with 5% BSA in Tris-buffered saline containing 0.1% (v/v) Tween 20 (TBST) for 1 h at room temperature and probed with primary antibodies against corresponding antigens overnight at 4 °C. Then, membranes were incubated with appropriate secondary antibodies, HRP-goat-anti-mouse (Abcam, ab6789) or HRP-goat-anti-rabbit (Abcam, ab6721), to bind the primary antibodies for 1 h at room temperature. The proteins were visualized by exposure machine (GE, Amersham Imager 680) using Pierce™ ECL Plus Western Blotting Substrate (Thermo Fisher Scientific, 32132) and the protein bands were quantitatively analyzed with ImageJ software.

The primary antibodies employed were: P53 (Proteintech, 60283-2-Ig), pSer46-P53 (CST, 2521), FOXO4 (Proteintech, 21535-1-AP), P21 (CST, 2947), P16 (CST, 18769), BAX (CST, 2772), BCL2 (CST, 3498), Ki-67 (Proteintech, 84192-4-RR), Lamin B (CST, 13435), Cleaved Caspase 3 (CST, 9664), γ-H2AX (Abcam, ab81299), and GAPDH (Abcam, ab8245) as an internal reference for normalizing protein expression levels.

### 2.4 SA-β-Gal stain

SA-β-Gal staining of cells and tissues was performed using a senescence-associated β-galactosidase staining kit (C0602, Beyotime Biotechnology, Shanghai, China), with all procedures strictly following the manufacturer’s instructions. For cells, they were first washed with PBS and fixed at room temperature for 15 min, followed by incubation with the staining solution at pH 6.0 overnight at 37 °C. For tissues, mice from different groups were euthanized by inhalation anesthesia. The thoracic aortas were surgically excised and carefully cleared of surrounding tissues. The aortas were then incubated in freshly prepared SA-β-Gal staining solution at pH 6.0 overnight at 37 °C, according to the manufacturer’s protocol. Subsequently, the tissues were cut open along one side and laid flat on glass slides. An anti-fade mounting medium was applied, and coverslips were placed to seal the samples. Staining results from both cells and tissues were observed and imaged using an Axio Observer 7 microscope (Carl Zeiss Meditec AG, Jena, Germany).

### 2.5 Ultrasound results analysis

Ultrasound aortic examinations were performed on mice subjected to different treatments. Mice were anesthetized by inhaling a mixture of 1 L/min oxygen and 1%–1.5% isoflurane via a mask, while maintaining normal respiration. Subsequently, a small-animal ultrasound system (Vevo F2, Canada) equipped with a linear 46-MHz probe was utilized to obtain long-axis M-mode aortic images and to assess aortic blood flow velocity using Doppler mode. Pulse wave velocity (PWV) was measured to reflect vascular elasticity, with higher PWV indicating greater arterial stiffness. All results were averaged from at least three consecutive measurements. All assessments were conducted by an experienced technician under blinded conditions.

### 2.6 Preparation of aortic tissue

After euthanizing mice from different groups using gas anesthesia, the thoracic aorta was surgically excised. The aorta was fixed in 4% fixative for 3 h, followed by thorough removal of surrounding tissues. The tissue underwent graded dehydration by sequential immersion in 30%, 50%, 70%, 90%, 100%, and 100% ethanol, each for 1 h. After dehydration, the tissue was cleared in xylene for 15 min, followed by infiltration with soft and hard paraffin wax, and finally embedded in paraffin. The paraffin-embedded tissue was sectioned into 5 μm-thick slices. The sections were baked in an oven at 65 °C for 2 h, then immersed in xylene for 5 min twice. Subsequently, the sections were deparaffinized through a graded series of ethanol (100%, 70%, 30%) and finally rinsed in PBS, each step lasting 5 min. The deparaffinized sections were used for immunofluorescence and hematoxylin and eosin staining.

### 2.7 Hematoxylin and eosin staining procedure

First, immerse the tissue sections in hematoxylin solution and stain at room temperature for 5 min. Then, quickly differentiate the sections by dipping them in 1% acid alcohol (1% HCl in ethanol) for 5 s. Immediately after differentiation, rinse the sections in running distilled water for 3 min to stop the differentiation. Next, immerse the sections in 0.5% ammonia water for 1–2 min until the tissue appears distinctly blue. Subsequently, immerse the sections in eosin solution and stain at room temperature for 2 min, followed by a brief rinse with distilled water. Since the tissue retains excess water after staining, dehydrate the sections by immersing them in absolute ethanol for 1 min. After dehydration, place the sections in an oven to ensure complete dryness by evaporating all ethanol. After removing the sections from the oven, add 1–2 drops of neutral mounting medium onto the center of each section. Gently apply a coverslip onto the section from one side using forceps. Finally, place the mounted slides flat in a well-ventilated area and allow them to dry naturally.

### 2.8 CCK-8 assay

HUVECs were evenly seeded into 96-well plates at a density of 1 × 10^4 cells per well, with a culture medium volume of 100 µL per well. The cells were incubated at 37 °C with 5% CO_2_ for 24 h to allow for proper attachment. According to the experimental design described in Section 2.2, the cells were then subjected to the respective treatments. After treatment, 10 µL of CCK-8 reagent was added to each well and gently mixed. The plate was then incubated at 37 °C with 5% CO_2_ for 1 h. Following incubation, the absorbance at 450 nm was measured for each well using a microplate reader.

### 2.9 Flow cytometric cell sorting analysis

Following the procedure described in Section 2.2, HUVEC cells were digested and collected using trypsin without EDTA. The cells were then washed with PBS buffer, and 1 × 10^6 cells were taken for each sample. According to the instructions of the Annexin V-FITC Apoptosis Detection Kit (Beyotime, C1062), 5 μL of Annexin V-FITC reagent was added and gently mixed, followed by the addition of 10 μL of propidium iodide staining solution, which was also gently mixed. The mixture was incubated at room temperature in the dark for 20 min, then immediately placed on ice for cooling. Finally, apoptosis analysis was performed using a flow cytometer (CytoFlex SRT, Beckman Coulter).

### 2.10 Liver and kidney function tests

After treatment according to the method described in Section 2.1, serum samples were collected from each group of mice. Liver function parameters were assessed using the ALT assay kit (Bestbio, BB-47448) and AST assay kit (Bestbio, BB-47449). Kidney function was evaluated using the urea assay kit (Beyotime, S0574) and the Amplex Red creatinine assay kit (Beyotime, S0291). All assays were performed strictly following the manufacturers’ instructions.

### 2.11 Separation and extraction of nuclear and cytoplasmic proteins

In this experiment, nuclear and cytoplasmic fractionation was performed using the Nuclear and Cytoplasmic Protein Extraction Kit (Beyotime, P0027) following the manufacturer’s instructions. Cells were first gently lysed with the cytoplasmic extraction buffer to release cytoplasmic components, and the supernatant was collected after centrifugation as the cytoplasmic protein fraction. The pellet containing nuclei was then treated with the nuclear extraction buffer to obtain nuclear proteins. The entire procedure was carried out on ice to preserve protein activity and stability. Finally, the purity of the fractions was confirmed by Western blot analysis using cytoplasmic marker protein GAPDH and nuclear marker protein Histone H3(HH3).

### 2.12 Immunofluorescence

The aorta (5 microns thick) was subjected to antigen retrieval by heating it in 10 mM citrate buffer (pH 6.0) at 95 °C for 10 min after decalcification and hydration. For endothelial cells, which were cultured in a confocal dish, they were fixed with paraformaldehyde at room temperature for 15 min. The samples were washed three times with PBS, each time for 5 min. Both endothelial cells and aortic sections were treated with 0.5% (v/v) Triton X-100 for 15 min, followed by three washes with PBS, each time for 5 min, and then blocked with 5% (v/v) bovine serum albumin (BSA, Sigma-Aldrich, B2064) at room temperature for 1 h. Subsequently, the samples were incubated overnight at 4 °C with primary antibody. After washing, the samples were incubated with secondary antibody. Finally, the cell nuclei were labeled with DAPI. Images were captured using a LSM 980 with Airyscan2 confocal microscope (Carl Zeiss Meditec AG, Jena, Germany).

The primary antibodies used are as follows: P53 (Proteintech, 60283-2-Ig), pSer46-P53 (CST, 2521), FOXO4 (Proteintech, 21535-1-AP), Ki-67 (D3B5) Rabbit mAb (Alexa Fluor® 647 Conjugate) (CST, 12075), P21 (CST, 14074), γ-H2AX (Abcam, ab2254).

The secondary antibodies used are as follows: Goat Anti-Mouse IgG H&L (Alexa Fluor® 488) (Abcam, ab150113), Goat Anti-Mouse IgG H&L (Alexa Fluor® 647) (Abcam, ab150115), Goat Anti-Rabbit IgG H&L (Alexa Fluor® 488) (Abcam, ab150077), Donkey Anti-Rabbit IgG H&L (Alexa Fluor® 647) (Abcam, ab150075).

### 2.13 DHE stain

For human umbilical vein endothelial cells (HUVECs), after different treatment groups, the cells were cultured in a glucose-free medium under hypoxic conditions for 3 h. Subsequently, 1 μM dihydroethidium (DHE) was added to the medium and further incubated for 30 min. Images were then acquired using an inverted microscope. For the aortic samples from aged mice, first, the aortas from different treatment groups were excised and other tissues were removed, leaving only the aortic endothelium. The aortic endothelium was then placed in a medium containing 1 μM DHE and incubated at 37 °C for 1 h. The staining results were visualized using an Axio Observer7 microscope (Carl Zeiss Meditec AG, Jena, Germany).

### 2.14 *In vitro* angiogenesis (tube formation) assay

The *in vitro* angiogenic activity of HUVECs (human umbilical vein endothelial cells) was determined by the Matrigel tube formation assay. After different groups of HUVECs are treated accordingly, they are seeded into 24-well plates pre-coated with 150 μL/well growth factor-reduced Matrigel (Corning, catalog number 354234) and incubated at 37 °C in a cell culture incubator for 12 h. Subsequently, the HUVECs are stained with a cell-permeable dye calcein AM (Corning, catalog number 354216) for 30 min, and then observed for capillary-like tubule formation using an inverted microscope. Tubular structures are defined as those with a length four times their width. Finally, the tube lengths from two wells are calculated and averaged using ImageJ software (National Institutes of Health, Bethesda, Maryland, United States). The staining results were visualized using an Axio Observer7 microscope (Carl Zeiss Meditec AG, Jena, Germany).

### 2.15 Real-time quantitative PCR

Total RNA was extracted from aortic tissue and endothelial cells using TRIzol reagent (Takara Bio Inc., 9108), following the manufacturer’s instructions. The RNA samples (1 ng) were reverse transcribed into cDNA using the Hiscript® III Reverse Transcriptase Kit (Vazyme, R223-01). RT-qPCR analysis was performed on a QuantStudio™ 3 Real-Time PCR Detection System using ChamQ Universal SYBR qPCR Master Mix (Vazyme, Q711-02) with specific primers. The relative expression levels of each gene were quantitated using the 2^−ΔΔCT^ method and normalized to the amount of endogenous glyceraldehyde-3-phosphate dehydrogenase (GAPDH). The sequences of specific primers used for RT-qPCR in this study are listed in Supplementary Table S1.

### 2.16 Co-IP

The FOXO4 co-immunoprecipitation assay was conducted using antibodies against FOXO4 and p53. Each sample group contained at least 10 million endothelial cells. Cells were lysed with 500 μL of RIPA lysis buffer containing 150 mM NaCl, 1% NP-40, 0.5% sodium deoxycholate, 0.1% SDS, 25 mM Tris-HCl, supplemented with protease and phosphatase inhibitors at a 1:100 dilution. The lysates were collected by scraping and then subjected to sonication for 1 min. Subsequently, lysates were centrifuged at 12,000 rpm for 15 min at 4 °C to obtain clear supernatants. The supernatants were incubated with Protein A/G Dynabeads pre-washed with ice-cold PBS and coupled to the respective antibodies. Incubation was carried out overnight (∼12 h) at 4 °C under gentle rotation. After incubation, immunocomplexes were retrieved using a magnetic stand, and the supernatant was discarded. The beads were washed three times for 5 min each with low-salt wash buffer containing 20 mM Tris-HCl, 1% Triton X-100, 2 mM EDTA, 150 mM NaCl, and 0.1% SDS. Between 1% and 10% of the lysate was retained as input control, and the remaining lysate was used for immunoprecipitation. Finally, the immunocomplexes were eluted with 20 μL of 2× loading buffer, boiled for denaturation, and subjected to SDS-PAGE.

### 2.17 TUNEL

Cell or tissue sections were fixed with 4% paraformaldehyde at room temperature for 15 min, followed by three washes with phosphate-buffered saline (PBS). Subsequently, the samples were permeabilized with 0.3% Triton X-100 in PBS at room temperature for 10 min. The samples were then incubated with the equilibration buffer provided in the TUNEL assay kit at room temperature for 10 min, followed by incubation with the reaction mixture containing terminal deoxynucleotidyl transferase and labeled dUTP. The samples were incubated in a humidified chamber at 37 °C in the dark for 60 min. After incubation, the samples were washed three times with PBS to remove excess reaction solution. Next, the nuclei were counterstained with DAPI to visualize all cells, and the slides were finally mounted with an anti-fade reagent. The staining results were observed and imaged using an Axio Observer 7 microscope (Carl Zeiss Meditec AG, Jena, Germany).

### 2.18 Statistical analysis

In this study, both cell and animal experiments were conducted using a blinded design. For the cell experiments, all cell samples were randomly coded by an independent third party, and experimenters conducted treatments and measurements without knowledge of the specific group assignments. In the animal experiments, animals were randomly grouped and coded by an independent individual, and both the experimenters and data analysts were blinded to the group allocations. The use of blinding minimized potential bias and enhanced the validity and reliability of the experimental results.

Data were analyzed using GraphPad Prism 8.0 software and presented as mean ± standard error of the mean (S.E.M.). Differences between samples were assessed using independent two-tailed t-tests or analysis of variance (ANOVA). For repeated measures data, two-way repeated measures ANOVA (2-way RM ANOVA) was employed. A p-value ≤0.05 was considered statistically significant. All experiments were conducted with a minimum of three replicates.

## 3 Results

### 3.1 FOXO4-DRI effectively improves vascular function in naturally aging mice

**FIGURE 1:**
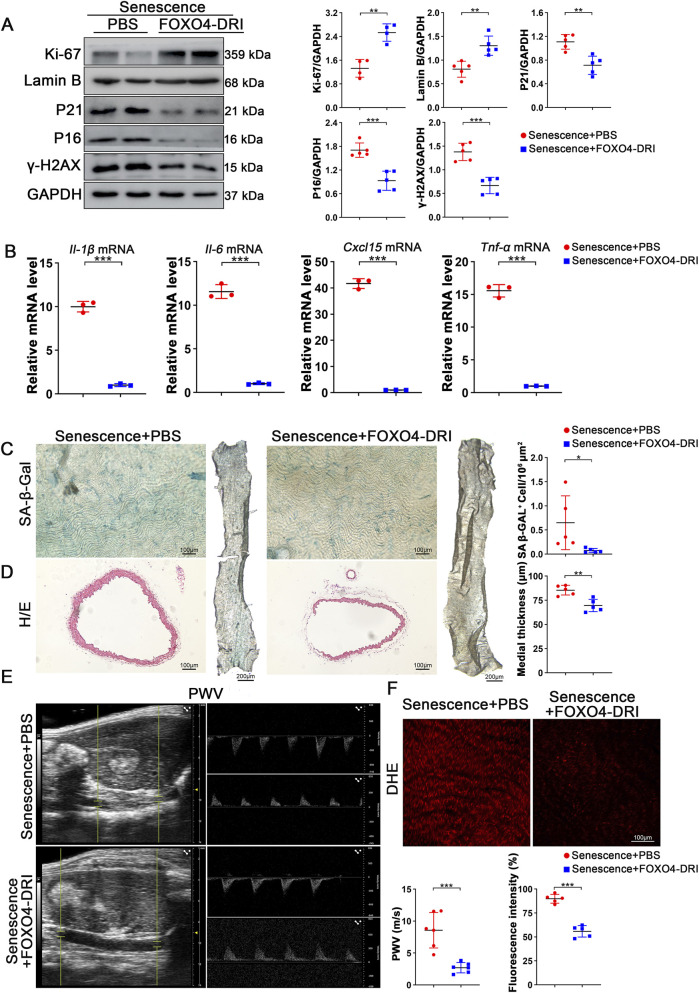
FOXO4-DRI can effectively maintain vascular function in naturally aging mice. **(A)** Western blot experiments were conducted and quantitatively analyzed to determine the changes in protein levels of Ki-67, Lamin B, P21, P16, and γ-H2AX in the aortas of naturally aged mice after FOXO4-DRI or PBS treatment. n ≥ 4 per group. **(B)** RT-qPCR analysis was performed to examine the mRNA levels of *Il-1β, Il-6, Cxcl15*, and *Tnf-α* in the aortas of naturally aged mice after FOXO4-DRI and PBS treatments. n = 3 per group. **(C)** Representative SA-β-Gal staining and quantitative analysis were performed to determine the number of positive cells in the aortas of naturally aged mice after FOXO4-DRI and PBS treatments. n = 5 per group. Scale bar = 100 μm. **(D)** Representative HE staining and quantitative analysis were performed to evaluate the thickness of the aortas in naturally aged mice after FOXO4-DRI and PBS treatments. n = 5 per group. Scale bar = 100 μm. **(E)** Color Doppler imaging and analysis were performed to detect and analyze the structural and blood flow conditions of the aortas in naturally aged mice after FOXO4-DRI and PBS treatments. n = 6 per group. **(F)** Representative DHE staining and quantitative analysis were performed to assess the ROS levels in the aortas of naturally aged mice after FOXO4-DRI and PBS treatments. n = 5 per group. Scale bar = 100 μm. Data are presented as mean ± SEM and analyzed using a two-tailed Student’s t-test. * indicates P < 0.05, ** indicates P < 0.01, *** indicates P < 0.001.

FOXO4-DRI has made great progress in anti-aging research, but there has been little research on it in the cardiovascular field, especially in the study of vascular aging. Therefore, it is particularly urgent to explore the role of FOXO4-DRI in vascular aging research. For this purpose, we treated normal aging mice with intraperitoneal injection of FOXO4-DRI at a dose of 5 mg/kg for 1 month. We then analyzed the aorta from the mice. Western blot analysis of thoracic aorta proteins showed that the classic aging markers P21 and P16 were significantly downregulated following FOXO4-DRI treatment, whereas the expression levels of the cell proliferation marker Ki-67 and Lamin B, a key protein involved in nuclear structural stability, were markedly upregulated (Figure 1A). We also found that the DNA damage marker γ-H2AX was reduced. We extracted the mRNA from the aorta of mice in different groups and performed RT-qPCR analysis. We found that the inflammatory factors, *Il-1β, Il-6, Cxcl15*, and *Tnf-α*, which are related to aging, were significantly decreased in the aging-related secretory phenotype (Figure 1B). We stained the aorta with SA-β-gal for analysis of the degree of vascular aging. We found that the number of SA-β-gal positive cells was reduced after FOXO4-DRI treatment (Figure 1C). Furthermore, H&E staining showed that compared to the aorta of normal aging mice, the aorta of mice treated with FOXO4-DRI was thinner, and the PWV value was lower in the ultrasound detection results, which means that the mice treated with FOXO4-DRI had better vascular elasticity and better vascular function than normal aging mice (Figures 1D,E). The DHE staining results also showed that FOXO4-DRI can effectively reduce the production of ROS in the vessels of aging mice (Figure 1F).

### 3.2 FOXO4-DRI effectively improves vascular function in prematurely aging mice

**FIGURE 2:**
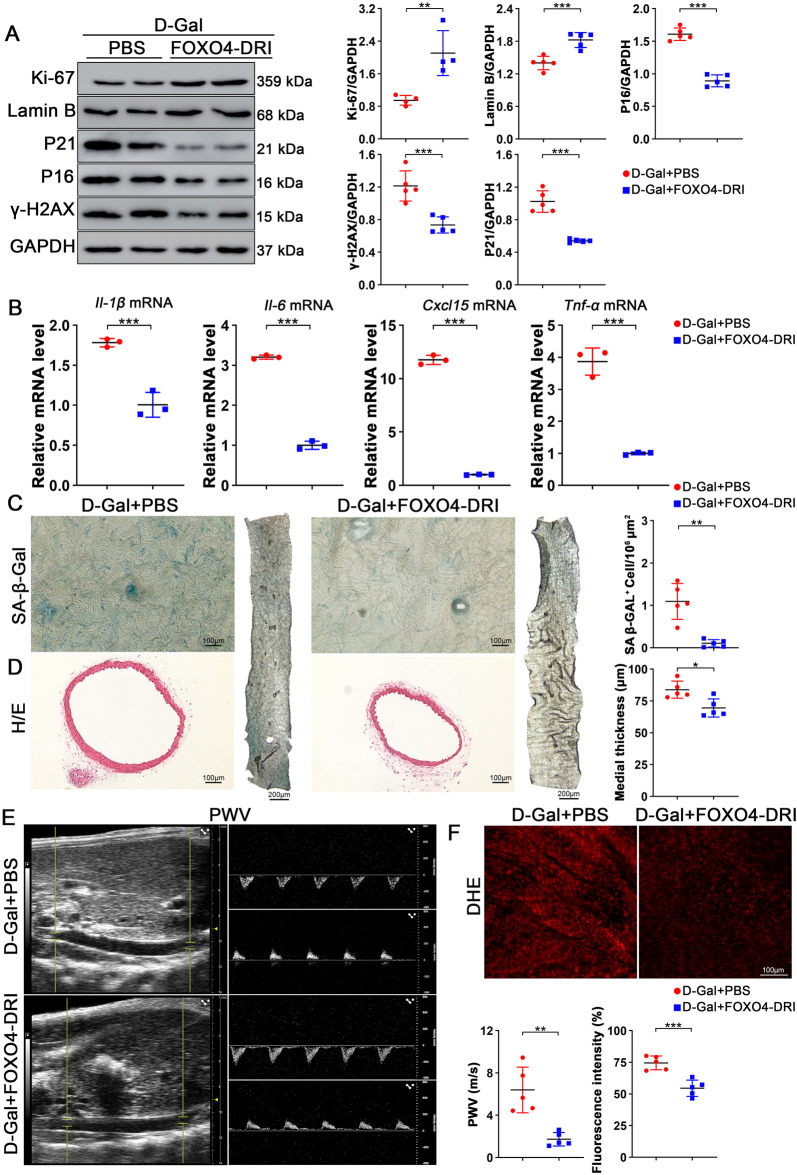
FOXO4-DRI can effectively maintain vascular function in progeroid mice. **(A)** Western blot experiments were conducted and quantitatively analyzed to determine the changes in protein levels of Ki-67, Lamin B, P21, P16, and γ-H2AX in the aortas of progeroid mice after FOXO4-DRI or PBS treatment. n ≥ 4 per group. **(B)** RT-qPCR analysis was performed to examine the mRNA levels of *Il-1β*, *Il-6*, *Cxcl15*, and *Tnf-α* in the aortas of progeroid mice after FOXO4-DRI and PBS treatments. n = 3 per group. **(C)** Representative SA-β-Gal staining and quantitative analysis were performed to determine the number of positive cells in the aortas of progeroid mice after FOXO4-DRI and PBS treatments. Scale bar = 100 μm. n = 5 per group. **(D)** Representative HE staining and quantitative analysis were performed to evaluate the thickness in the aortas of progeroid mice after FOXO4-DRI and PBS treatments. Scale bar = 100 μm. n = 5 per group. **(E)** Color Doppler imaging and analysis were performed to detect and analyze the structural and blood flow conditions in the aortas of progeroid mice after FOXO4-DRI and PBS treatments. n = 5 per group. **(F)** Representative DHE staining and quantitative analysis were performed to assess the ROS levels in the aortas of progeroid mice after FOXO4-DRI and PBS treatments. n = 5 per group. Scale bar = 100 μm. Data are presented as mean ± SEM and analyzed using a two-tailed Student’s t-test. * indicates P < 0.05, ** indicates P < 0.01, *** indicates P < 0.001.

To further explore the role of FOXO4-DRI in aged mice, we constructed prematurely aging mouse model by administering D-galactose (200 mg/kg) intraperitoneally for 8 weeks. By extracting aortic tissue from the mice and performing WB analysis, we found that the expression levels of P53 and FOXO4 were both elevated (Supplementary Figure S1A). Immunofluorescence analysis demonstrated FOXO4 nuclear accumulation in aortic tissues of D-galactose-induced aging mice (Supplementary Figure S1B). These results indicate that D-galactose successfully induced an aging model in mice. In our experimental design, we established a D-galactose-induced aging mouse model and injected the experimental group mice with FOXO4-DRI for 1 month, while the control group was injected with equal amounts of PBS. By analyzing the aortic tissue, we found that FOXO4-DRI treatment significantly increased the expression levels of Ki-67 and Lamin B, while reducing the expression of P21, P16, and γ-H2AX, similar to the results obtained from naturally aged mice (Figure 2A). Analysis of mRNA from the aortas of different groups showed that FOXO4-DRI effectively inhibited the increase of pro-inflammatory factors such as *Il-1β*, *Il-6*, *Cxcl15*, and *Tnf-α* (Figure 2B). Through SA-β-gal staining, we found that FOXO4-DRI also reduced the number of positive cells in the aorta of D-galactose induced aged mice (Figure 2C). H&E staining results further confirmed that the aortic wall thickness of mice treated with FOXO4-DRI was significantly thinner, and ultrasound examination results showed that the PWV of the aorta was slowed down after FOXO4-DRI treatment, indicating better vascular elasticity and significantly improved vascular function compared to the control group (Figures 2D,E). Additionally, in the DHE staining results, we observed that FOXO4-DRI treatment effectively reduced the ROS levels in the aorta of aged mice (Figure 2F). These results collectively demonstrate that FOXO4-DRI can effectively improve the aortic function of aged mice.

### 3.3 Hypoxic-glucose deprivation conditions can induce senescence in endothelial cells

Unlike the previously mentioned natural aging models and progeria models, there is another common but often overlooked endothelial cell aging model: hypoxia-induced endothelial cell senescence. This type of endothelial cell senescence is particularly prevalent in various cardiovascular diseases, including atherosclerosis, hypertension, diabetes, chronic ischemic diseases, and cerebrovascular diseases. These conditions are all closely related to hypoxia-induced endothelial cell senescence. Therefore, establishing a hypoxia-induced endothelial cell senescence model is crucial for studying these diseases.

**FIGURE 3:**
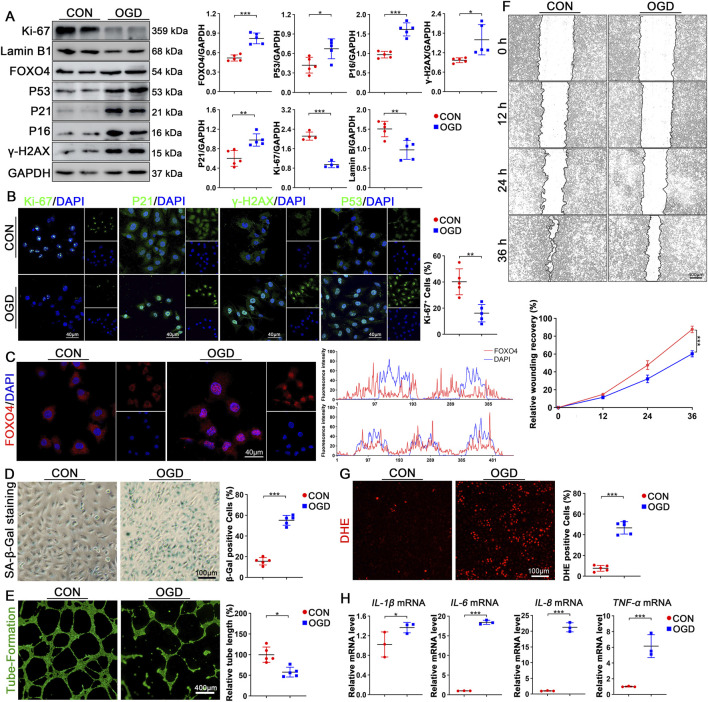
Glucose and oxygen deprivation can induce endothelial cell senescence. **(A)** Western blot analysis was performed to quantify the changes in protein levels of Ki-67, Lamin B, FOXO4, P53, P21, P16, and γ-H2AX in HUVECs following OGD treatment, with n ≥ 4 per group. **(B)** Representative immunofluorescence staining was conducted to analyze the content and localization changes of Ki-67, P21, γ-H2AX, and P53 different groups of HUVECs, with n = 5 per group. **(C)** Subcellular localization of FOXO4 in different groups of HUVECs was analyzed through representative immunofluorescence staining (FOXO4 in red, DAPI in blue), with n = 5 per group, scale bar = 40 μm. **(D)** Representative SA-β-GAL staining and quantification were performed to evaluate the number of positive cells in HUVECs after OGD treatment, with n = 5 per group, scale bar = 100 μm. **(E)** Tube formation assays analyzed relative tube lengths in different groups following OGD treatment, with n = 5 per group, scale bar = 400 μm. **(F)** Scratch assays were conducted to assess endothelial cell migration coverage at 0, 12, 24, and 36 h across different groups, with n = 5 per group, scale bar = 400 μm. Data are represented as mean ± SEM. Statistical analysis assessed by 2-way RM ANOVA **(G)** Representative DHE staining and quantification evaluated the number of DHE-positive cells in different groups, with n = 5 per group, scale bar = 100 μm. **(H)** RT-qPCR analysis was performed in HUVECs following OGD treatment to measure the mRNA levels of *IL-6*, *IL-8*, *IL-1β*, and *TNF-α*, with n = 3 per group. Data are presented as mean ± SEM and analyzed using a two-tailed Student’s t-test. * indicates P < 0.05, ** indicates P < 0.01, *** indicates P < 0.001.

After 3 h of OGD treatment in HUVECs, WB results showed that the expression of Ki-67 and Lamin B decreased, while the expression of P53, P21, P16, and γ-H2AX significantly increased (Figure 3A). After OGD treatment, the number of Ki-67-positive endothelial cells significantly decreased, while the intracellular expression levels of senescence markers P21, P53, and the DNA damage indicator γ-H2AX were markedly elevated (Figure 3B). Additionally, OGD treatment resulted in a significant increase in FOXO4 expression (Figure 3C), and the accumulation of FOXO4 in the nucleus was more evident through immunofluorescence technology. To further verify whether OGD treatment could cause endothelial cell senescence, β-galactosidase (SA-β-gal) staining was performed on the endothelial cells treated with OGD. The results showed that the proportion of SA-β-gal positive cells after OGD treatment was significantly higher than that in the control group (Figure 3D). Meanwhile, in the functional tests, we observed that the tubular formation ability of endothelial cells was significantly decreased after OGD treatment (Figure 3E). In the cell migration experiment, the rate of scratch healing was also significantly slower than that of the control group, indicating that OGD treatment caused damage to endothelial cell function (Figure 3F). Furthermore, dihydroethidine (DHE) staining showed that the proportion of DHE-positive cells after OGD treatment was higher than that in the control group, suggesting that the level of reactive oxygen species (ROS) was increased (Figure 3G). In the detection of pro-inflammatory factor secretion, we also found that OGD treatment promoted the expression of pro-inflammatory factors, indicating that the inflammatory response was enhanced (Figure 3H).

In summary, these experimental results collectively confirmed the damage and aging characteristics of endothelial cells caused by OGD treatment.

### 3.4 FOXO4-DRI alleviates OGD-induced endothelial cell senescence

**FIGURE 4:**
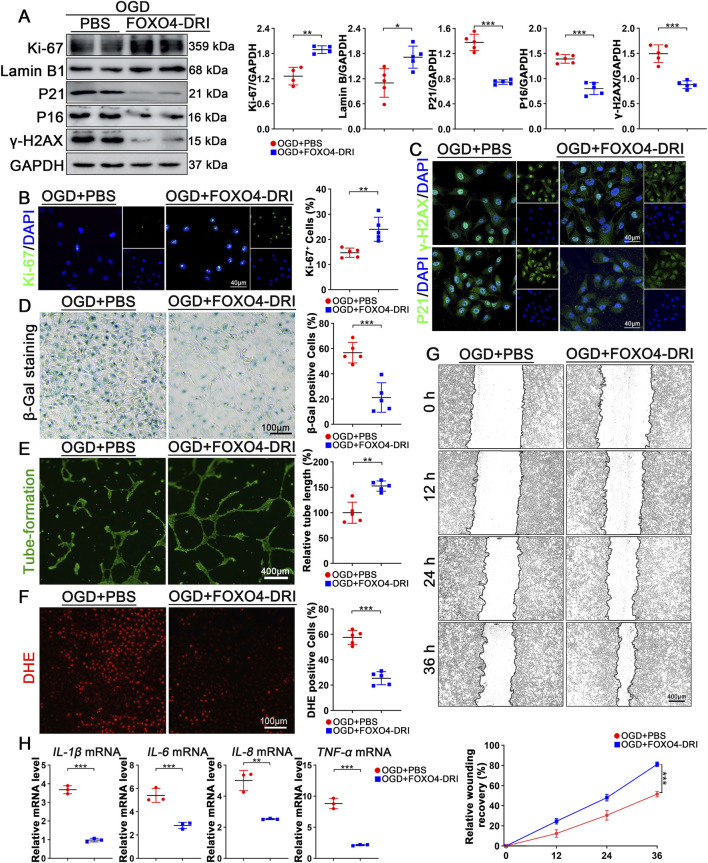
FOXO4-DRI can alleviate endothelial cell senescence induced by OGD. **(A)** Western blot analysis was conducted to quantify the changes in protein levels of Ki-67, Lamin B, P21, P16, and γ-H2AX in senescent HUVECs following treatment with FOXO4-DRI or PBS, with n ≥ 4 per group. **(B)** Representative immunofluorescence analysis (green for Ki-67, blue for DAPI) was performed to assess the number of Ki-67 positive HUVECs in different groups, with n = 5 per group and scale bar = 40 μm. **(C)** Fluorescent staining was carried out to analyze the content and localization changes of P21 and γ-H2AX in different groups, with n = 5 per group and scale bar = 40 μm. **(D)** Representative SA-β-GAL staining and quantification were performed to evaluate the number of positive cells in different groups, with n = 5 per group and scale bar = 100 μm. **(E)** Tube formation assays were conducted to analyze relative tube lengths in different groups, with n = 5 per group and scale bar = 400 μm. **(F)** Representative DHE staining and quantification were carried out to evaluate the number of DHE-positive cells in different groups, with n = 5 per group and scale bar = 100 μm. **(G)** Scratch assays were performed to assess endothelial cell migration coverage at 0, 12, 24, and 36 h in different groups, with n = 5 per group and scale bar = 400 μm. Data are represented as mean ± SEM. Statistical analysis assessed by 2-way RM ANOVA. **(H)** RT-qPCR analysis was performed to detect mRNA levels of *IL-6*, *IL-8*, *IL-1β*, and *TNF-α* in different groups, with n = 3 per group. Data are presented as mean ± SEM and analyzed using a two-tailed Student’s t-test. * indicates P < 0.05, ** indicates P < 0.01, *** indicates P < 0.001.

We investigated the effects of FOXO4-DRI on OGD (oxygen-glucose deprivation)-induced senescent endothelial cells by treating them with PBS and FOXO4-DRI. Western blot (WB) results revealed a significant increase in the expression of Ki-67 and Lamin B, while the protein levels of P21, P16, and γ-H2AX decreased following FOXO4-DRI treatment (Figure 4A). Immunofluorescence staining also showed a marked increase in the number of Ki-67-positive cells and a decrease in the nuclear content of γ-H2AX and P21 after FOXO4-DRI treatment (Figures 4B,C). SA-β-gal staining demonstrated a notable reduction in the number of SA-β-gal-positive cells post-FOXO4-DRI treatment compared to the PBS group (Figure 4D). Endothelial cell tube formation was significantly impaired after OGD treatment, but showed partial recovery following FOXO4-DRI treatment (Figure 4E). Additionally, FOXO4-DRI reduced the DHE-positive cell count, indicating decreased ROS levels under OGD conditions (Figure 4F). The migration rate of endothelial cells, an important functional indicator, was found to be accelerated after FOXO4-DRI treatment (Figure 4G). SASP (senescence-associated secretory phenotype) results revealed that FOXO4-DRI downregulates the levels of *IL-1β*, *IL-6*, *IL-8*, and *TNF-α* (Figure 4H). These findings collectively demonstrate that FOXO4-DRI alleviates OGD-induced endothelial cell senescence and restores functional damage caused by OGD.

### 3.5 FOXO4-DRI inhibits FOXO4-P53 binding and promotes P53 phosphorylation

**FIGURE 5:**
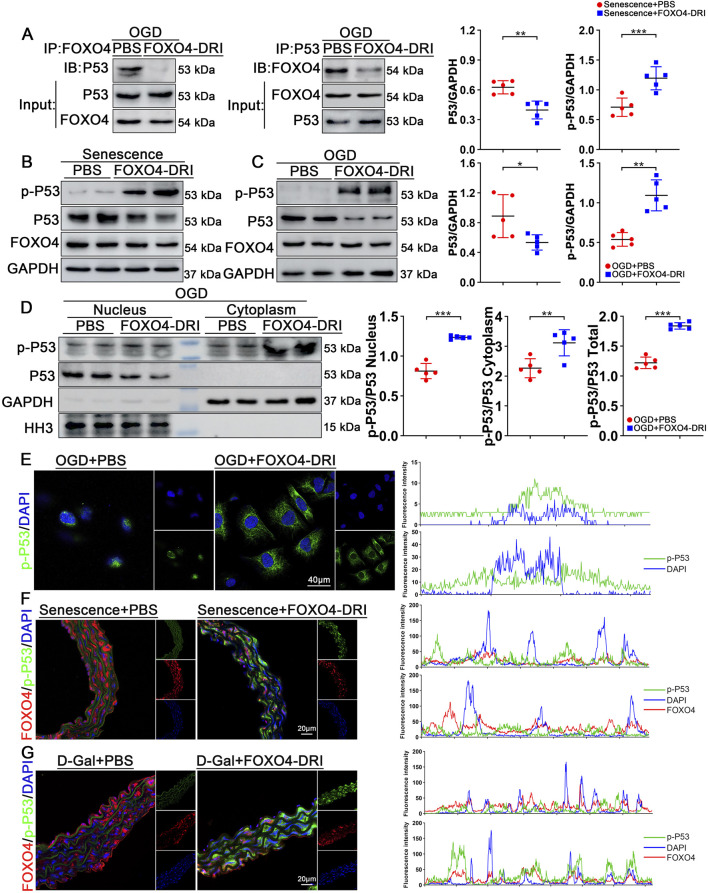
FOXO4-DRI can block the interaction between P53 and FOXO4. **(A)** The FOXO4-P53 interaction was analyzed through co-immunoprecipitation in OGD-treated HUVECs, in different groups, with n = 3 per group. **(B)** Western blot analysis quantified changes in protein levels of pSer46-P53 and P53 in different groups, with n = 5 per group. **(C)** Western blot analysis quantified changes in protein levels of pSer46-P53 and P53 in different groups, with n = 5 per group. **(D)** Western blot analysis was performed to examine the distribution of pSer46-P53 and P53 in the cytoplasm and nucleus of HUVECs in different groups. With n = 5 per group. **(E)** Subcellular localization of pSer46-P53 in different groups of HUVECs was analyzed using representative immunofluorescence staining (pSer46-P53 in green, DAPI in blue), with n = 5 per group, scale bar = 40 μm. **(F)** Immunofluorescence detection and analysis of subcellular localization of pSer46-P53 and FOXO4 in aortic sections of naturally aged mice (pSer46-P53 in green, FOXO4 in red, DAPI in blue), with n = 5 per group, scale bar = 20 μm. **(G)** Immunofluorescence detection and analysis of subcellular localization of pSer46-P53 and FOXO4 in aortic sections of progeroid mice (pSer46-P53 in green, FOXO4 in red, DAPI in blue), with n = 5 per group, scale bar = 20 μm. Data are presented as mean ± SEM and analyzed using a two-tailed Student’s t-test. *P < 0.05, **P < 0.01, ***P < 0.001.

To further elucidate the specific mechanism of FOXO4-DRI in anti-aging, we induced senescence in endothelial cells under OGD conditions and treated these cells either PBS or FOXO4-DRI. To validate the impact of FOXO4-DRI on the P53 signaling pathway in senescent endothelial cells, Co-IP experiments were performed. The results showed a significant decrease in the binding of FOXO4 to P53 after FOXO4-DRI treatment (Figure 5A). Subsequently, Western Blot (WB) analysis detected a decrease in total P53 expression but an increase in phosphorylated P53 (p-P53) expression in OGD-induced senescent endothelial cells post FOXO4-DRI treatment (Figures 5B,C). We separated nuclear and cytoplasmic proteins and calculated the ratio of p-P53 to total P53 in each fraction. The phosphorylation status of P53 was also assessed in whole-cell lysates. The results showed that after FOXO4-DRI treatment, the p-P53/P53 ratio significantly increased in the nucleus, cytoplasm, and total protein samples (Figure 5D). Additionally, immunofluorescence experiments indicated that under senescent conditions, p-P53 primarily accumulated in the cell nucleus (Figure 5E). Furthermore, WB analysis was conducted on extracts from the aortas of aging mice, showing consistent results with *in vitro* experiments: increased expression of p-P53 and decreased expression of total P53 after FOXO4-DRI treatment. Similar observations were made in aortic tissue slices, where FOXO4-DRI treatment led to increased expression of p-P53, mainly localized in the cytoplasm (Figures 5F,G). In summary, FOXO4-DRI exerts its anti-aging effects by inhibiting the binding of FOXO4 to P53, promoting P53 phosphorylation, and facilitating the translocation of p-P53 from the nucleus to the cytoplasm for its functional role.

### 3.6 FOXO4-DRI promotes apoptosis in senescent cells

**FIGURE 6:**
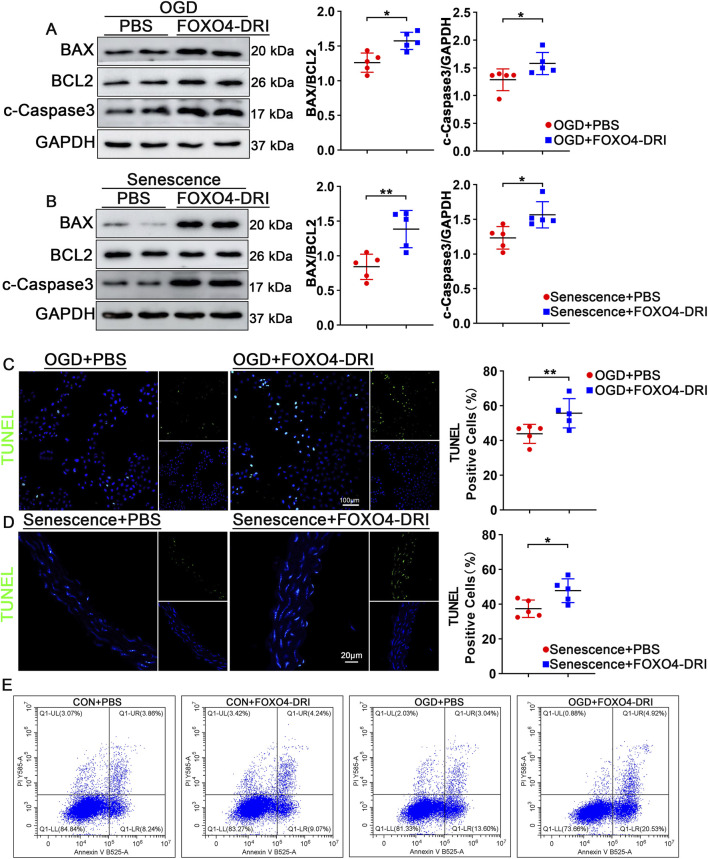
FOXO4-DRI promotes apoptosis in senescent cells. **(A)** Western blot analysis quantified changes in protein levels of BAX, Bcl2, and c-Caspase3 in different groups, with n = 5 per group. **(B)** Western blot analysis assessed changes in protein levels of BAX, Bcl2, and c-Caspase3 in the aortas of naturally aged mice after FOXO4-DRI or PBS treatment, with n = 5 per group. **(C)** Representative TUNEL staining and quantitative analysis were conducted to determine the number of positive cells in the aortas of naturally aged mice after FOXO4-DRI and PBS treatments, with n = 5 per group and scale bar = 100 μm. **(D)** Representative TUNEL staining and quantification evaluated the number of TUNEL-positive cells across different groups with n = 5 per group and scale bar = 20 μm. **(E)** Flow cytometry analysis was used to quantitatively assess the number of Annexin V-positive cells in different groups, with a sample size of n = 5 per group. Data are presented as mean ± SEM and analyzed using a two-tailed Student’s t-test. *P < 0.05, **P < 0.01, ***P < 0.001.

As established in prior research, FOXO4-DRI competitively binds to p53 with high affinity, thereby disrupting its nuclear interaction with FOXO4. This leads to the release and cytoplasmic translocation of p53, which can subsequently activate transcription-independent pro-apoptotic signaling (Clara et al., 2024; Kong et al., 2025). To further validate whether FOXO4-DRI can induce apoptosis in senescent cells, we conducted the following experiments. In senescent endothelial cells induced by OGD, treatment with FOXO4-DRI led to a significant increase in the expression of BAX and cleaved caspase-3 (c-Caspase-3), while the expression of BCL2 notably decreased. Similar changes were observed in the aortic tissues of aging mice (Figures 6A,B). To assess the extent of apoptosis, we performed TUNEL staining experiments. The results showed a significant increase in the number of TUNEL-positive points in senescent endothelial cells treated with FOXO4-DRI. The same phenomenon was observed in the aortic tissues of aging mice after FOXO4-DRI treatment (Figures 6C,D). The results of flow cytometry also demonstrated that, as detected by Annexin staining, FOXO4-DRI treatment significantly promoted apoptosis in senescent cells (Figure 6E). To further explore the clinical application potential of FOXO4-DRI, we conducted liver and kidney function assessments in FOXO4-DRI-treated mice. Specifically, serum levels of alanine aminotransferase (ALT) and aspartate aminotransferase (AST) were measured to evaluate liver function, while blood urea nitrogen (BUN) and creatinine were measured to assess kidney function. Additionally, the CCK-8 assay was used to evaluate the effect of FOXO4-DRI treatment on cell viability. The results showed no significant differences in any of the indicators, indicating that FOXO4-DRI treatment did not adversely affect liver or kidney function or cell viability in mice (Supplementary Figures S2A–C). These findings indicate that FOXO4-DRI effectively promotes apoptosis in senescent cells. Overall these results confirm that FOXO4-DRI promotes apoptosis in senescent endothelial cells by inhibiting FOXO4-P53 binding and phosphorylating P53.

## 4 Discussion

**FIGURE 7:**
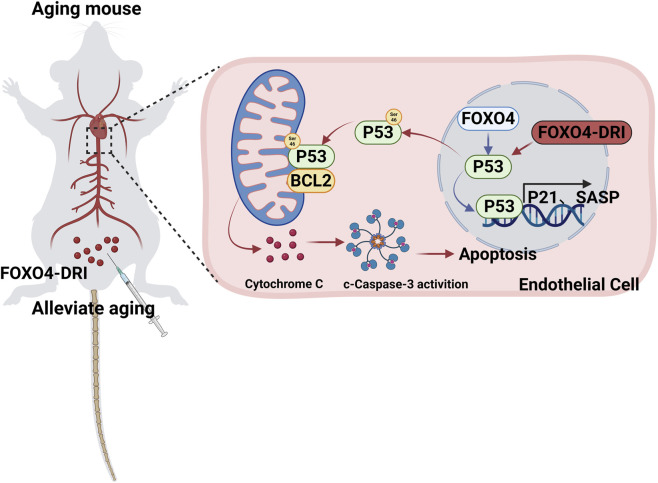
Schematic illustration of FOXO4-DRI in alleviating endothelial cell senescence aging extends cell survival. This study demonstrates that FOXO4-DRI mitigates senescence by inhibiting the FOXO4-P53 interaction, promoting P53 phosphorylation, and inducing apoptosis in senescent cells, thereby alleviating aging-related symptoms. Content produced with BioRender; license VN28TABHO4.

In this study, we reveal the beneficial effects of FOXO4-DRI on aortic vessels under various aging models. Our findings indicate that FOXO4-DRI promotes apoptosis in senescent cells by disrupting the FOXO4-P53 interaction, thereby releasing and phosphorylating P53, which then translocates to the cytoplasm. This mechanism effectively alleviates vascular aging and maintains normal vascular function (Figure 7).

Endothelial cell aging is closely associated with the progression of various diseases, potentially exacerbating the senescence of surrounding cells and worsening pathological processes (Hwang et al., 2022). While immune cells can recognize and eliminate senescent cells, many of these cells can evade immune detection in aging and disease states, allowing them to persist and negatively impact the function of neighboring cells (Augustin and Koh, 2024; Xian et al., 2021; Camille et al., 2021). Therefore, promoting the apoptosis of senescent endothelial cells and improving the microenvironment of aged blood vessels is crucial for maintaining vascular function.

Compared to normal aging and D-GAL-induced premature aging models, research on endothelial cell senescence induced by hypoxia under pathological conditions remains scarce. This aging mechanism is closely related to various common diseases, including atherosclerosis, hypertension, chronic obstructive pulmonary disease, diabetes, and neurodegenerative disorders (Liu et al., 2022; Ying et al., 2024). Such diseases often arise from impaired vascular function, leading to severe complications such as myocardial infarction and cerebrovascular accidents (Prakash Babu et al., 2019; Sun et al., 2020). Therefore, exploring strategies to improve endothelial cell function under these disease conditions is particularly important. In our study, FOXO4-DRI was found to effectively promote apoptosis in senescent cells by blocking the FOXO4-P53 interaction and facilitating P53 phosphorylation. This process not only slows down hypoxia-induced endothelial cell senescence but also aids in restoring damaged endothelial cell function, offering new insights and potential intervention strategies for related diseases.

Current senolytic agents are primarily employed in cancer therapies, such as doxorubicin, trametinib, and cell cycle-targeting drugs like palbociclib (Lea et al., 2022; Marcus et al., 2018). However, these agents often lack selectivity, potentially causing unnecessary damage and apoptosis in normal cells (Marcus et al., 2018; Tao et al., 2016). In contrast, FOXO4-DRI leverages the elevated expression of FOXO4 and P53 in senescent cells by specifically blocking the interaction between FOXO4 and P53 to promote apoptosis via the P53 signaling pathway (Tao et al., 2016; Emmanuelle et al., 2023; Paul and Anton, 2017). Thus, FOXO4-DRI selectively induces apoptosis in senescent cells without adversely affecting normal cells, providing new strategies and approaches to address the accumulation of senescent cells.

Although studies have shown that FOXO4-DRI can block the binding of P53 to FOXO4, thereby promoting the apoptosis of senescent cells, the effect of FOXO4-DRI is not limited to alleviating the aging of certain organs; it can resist overall individual aging (Yanqing et al., 2024; Xigang et al., 2024; Deryabin Pavel et al., 2022; Zhang et al., 2020). Therefore, we need to identify commonalities among different organs to truly reveal the effects of FOXO4-DRI in anti-aging. Endothelial cell senescence is a significant characteristic of all aging phenomena, and many different diseases are closely related to vascular endothelium (Zoltan et al., 2018; Angela et al., 2018). The initial stages of individual aging are also closely linked to vascular endothelial senescence (Brandes Ralf et al., 2005). Thus, by examining this angle, we aim to reveal the mechanism by which FOXO4-DRI alleviates individual aging, providing a new perspective for the treatment of aging using FOXO4-DRI. It is important to emphasize that although certain research progress has been made, our work is still at the preclinical stage and remains some way from clinical application; further in-depth studies are needed in the future to verify its safety and efficacy.

In summary, FOXO4 plays a critical role in multiple diseases, and studies have shown that endothelial cell senescence accompanies these conditions. Our research has demonstrated that FOXO4-DRI can improve vascular function by alleviating endothelial cell senescence, providing a novel therapeutic approach for related diseases.

## Data Availability

The raw data supporting the conclusions of this article will be made available by the authors, without undue reservation.
