# Supplementary material for: FOXO4-DRI regulates endothelial cell senescence via the P53 signaling pathway
Source: Front Bioeng Biotechnol. 2026 Jan 15;13:1729166. doi: 10.3389/fbioe.2025.1729166 (PMC12852416; doi:10.3389/fbioe.2025.1729166)
Supplement: Supplementary file 1 [file Supplementaryfile1.doc]

**Supplementary information**

**FOXO4-DRI regulates endothelial cell senescence via the P53 signaling pathway.**

Zhicheng Hu^2, 3†^, Fan Li^2, 3†^, Chunyi Hu^1, 2^,, Qiongdan Shan^2^, Zhouhao Tang^2^, Meifan Jiang^2, 3^, Xiaojing Yi^2^, Xixi Chen^4^, Litai Jin^2, 5^, Xu Wang^2*^, Yang Wang^1*^

**Author names and institutional affiliations**

^1^Department of Histology and Embryology, School of Basic Medical Sciences, Wenzhou Medical University, Wenzhou, P.R. China.

^2^School of Pharmaceutical Science, Wenzhou Medical University, Wenzhou, 325000, P.R. China.

^3^Oujiang Laboratory (Zhejiang Lab for Regenerative Medicine, Vision and Brain Health), School of Pharmaceutical Science, Wenzhou Medical University, Wenzhou, P.R. China.

^4^Department of pharmacy, Taizhou Central Hospital, Taizhou, Zhejiang, China.

^5^Ningbo Key Laboratory of Skin Science, Ningbo College of Health Sciences, Ningbo, 315000, P.R. China.

^†^ These authors contributed equally.

***Address** **correspondence to:**

Yang Wang, Department of Histology and Embryology, School of Basic Medical Sciences, Wenzhou Medical University, Wenzhou, P.R. China. E-mail: [yw1867@126.com](mailto:yw1867@126.com)

Xu Wang, School of Pharmaceutical Science, Wenzhou Medical University, Wenzhou, 325000, P.R. China. E-mail: [wang_xu2003@163.com](mailto:wang_xu2003@163.com)

**
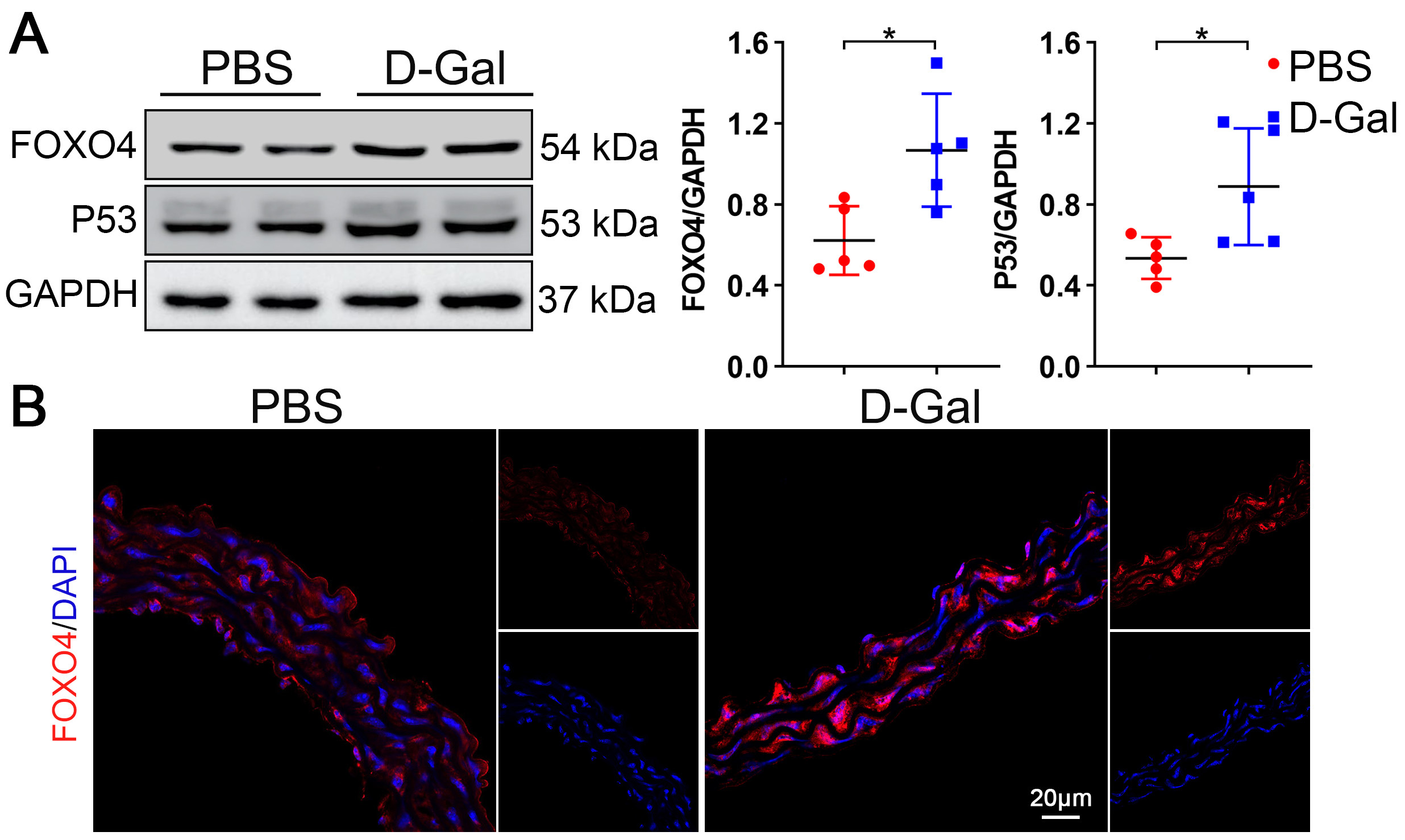
**

**Supplement Figure 1. D-Gal-induced endothelial cell senescence.**

(A) Western blot was performed and quantitatively analyzed to determine the protein levels of FOXO4 and P53 in the aorta tissues from the D-Gal or PBS group. n=5 per group. (B) Representative immunofluorescence staining analysis of FOXO4 protein expression in the aorta tissues from the D-Gal or PBS group. Scale bar=20 μm. Data represent means±SEM. Two-tailed Student’s t-test. * indicates P<0.05.


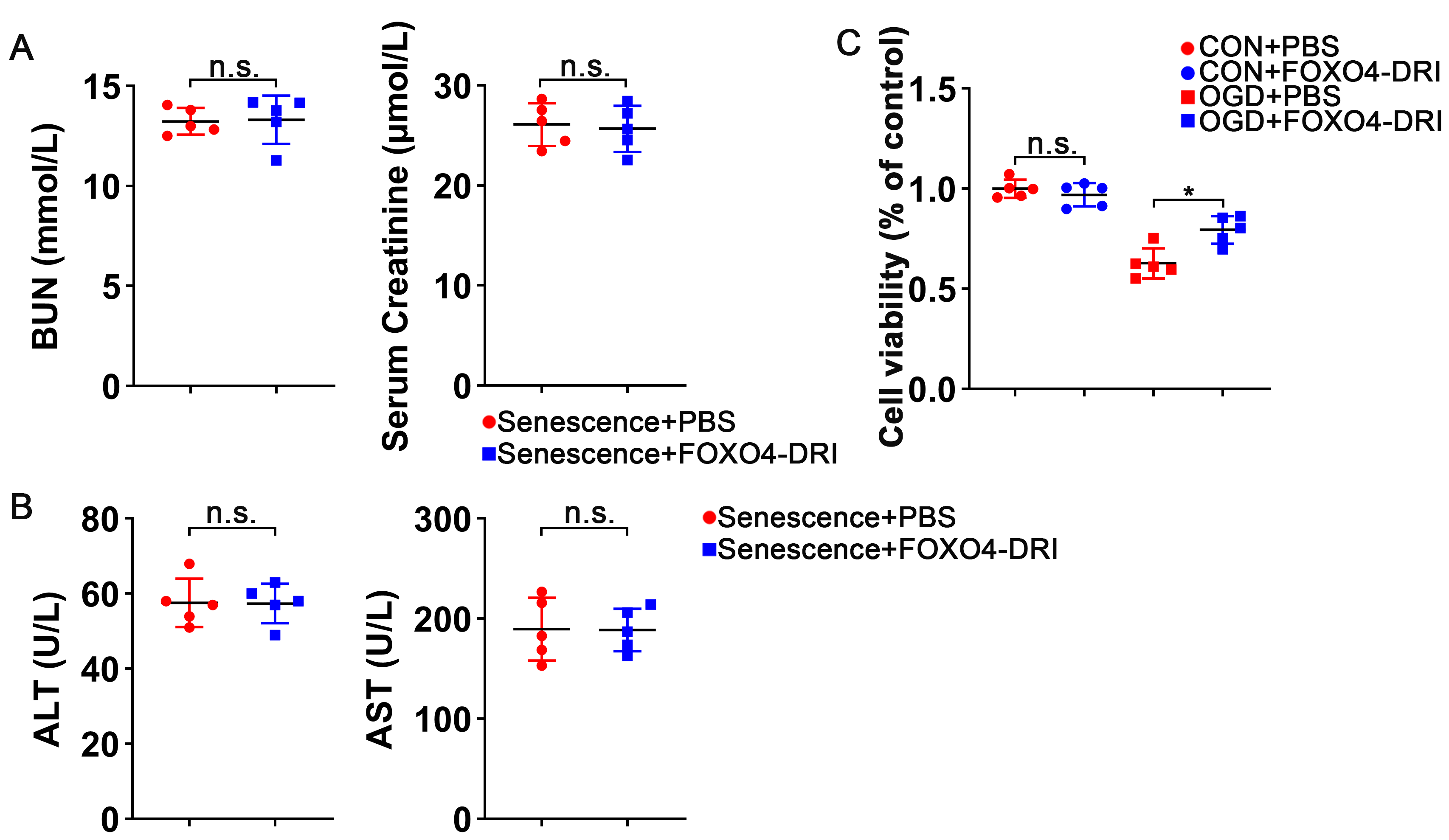


**Supplement Figure 2. Effects of FOXO4-DRI on liver, kidney, and cell viability.**

(A) Aged mice were injected with PBS or FOXO4-DRI, after which serum samples were collected for analysis of blood urea nitrogen (BUN) and serum creatinine levels. n=5 per group. (B) Aged mice were injected with PBS or FOXO4-DRI, after which serum samples were collected for analysis of ALT and AST levels. n=5 per group. (C) After incubation with PBS or FOXO4-DRI for 24 hours, the senescent group was cultured in glucose-free medium under hypoxic conditions for 3 hours; meanwhile, the control group was treated with CCK-8 reagent at the same time point and incubated for an additional 1 hour to assess cell viability. n=5 per group. (A)(B)Data represent means±SEM. Two-tailed Student’s t-test. (C) Data represent means ± SEM and were analyzed by one-way ANOVA.* indicates P<0.05.

**Supplementary Table 1. Detailed primers used in the RT-PCR.**

| Primer | Forward | Reverse |
| --- | --- | --- |
| *TNF-α(Human)* | 5'- GAGGCCAAGCCCTGGTATG -3' | 5'- CGGGCCGATTGATCTCAGC -3' |
| *IL-1β (Human)* | 5'-ATGATGGCTTATTACAGTGGCAA-3' | 5'- GTCGGAGATTCGTAGCTGGA -3' |
| *IL-6 (Human)* | 5'-ACTCACCTCTTCAGAACGAATTG-3' | 5'- CCATCTTTGGAAGGTTCAGGTTG - 3' |
| *CXCL-8 (Human)* | 5'- TTTTGCCAAGGAGTGCTAAAGA -3' | 5'- AACCCTCTGCACCCAGTTTTC -3' |
| *GAPDH (Human)*  *Tnf-α(Mouse)*  *Il-1β (Mouse)*  *Il-6 (Mouse)*  *CXCL-15 (Mouse)*  *Gapdh (Mouse)* | 5'- TGTGGGCATCAATGGATTTGG -3'  5'- CAGGCGGTGCCTATGTCTC -3'  5'- GAAATGCCACCTTTTGACAGTG -3'  5'- CTGCAAGAGACTTCCATCCAG -3'  5'- TCGAGACCATTTACTGCAACAG -3'  5'- AGGTCGGTGTGAACGGATTTG -3' | 5'- ACACCATGTATTCCGGGTCAAT -3'  5'- CGATCACCCCGAAGTTCAGTAG -3'  5'- TGGATGCTCTCATCAGGACAG -3'  5'- AGTGGTATAGACAGGTCTGTTGG -3'  5'- CATTGCCGGTGGAAATTCCTT -3'  5'- TGTAGACCATGTAGTTGAGGTCA -3' |
